# A pilot study to assess the influence of infiltrated stormwater on groundwater: Hydrology and trace organic contaminants

**DOI:** 10.1002/wer.10690

**Published:** 2022-02-04

**Authors:** Sarah M. Elliott, Richard L. Kiesling, Andrew M. Berg, Heiko L. Schoenfuss

**Affiliations:** ^1^ Upper Midwest Water Science Center U.S. Geological Survey Mounds View Minnesota USA; ^2^ Department of Biological Sciences St. Cloud State University St. Cloud Minnesota USA

**Keywords:** green infrastructure, organic contaminants, pesticides, pharmaceuticals, underground infiltration basin, urban stormwater

## Abstract

Underground infiltration basins (UIBs) mimic the natural hydrologic cycle by allowing stormwater to recharge local groundwater aquifers. However, little is known about the potential transport of organic contaminants to receiving groundwater. We conducted a pilot study in which we collected paired grab samples of stormwater runoff flowing into two UIBs (inflow) and shallow groundwater adjacent to the UIBs. Samples were collected coincident with three rain events and analyzed for volatile organic compounds, semi‐volatile organic compounds, pharmaceuticals, and pesticides. Few contaminants were detected in groundwater, compared with inflow, and groundwater concentrations were typically an order of magnitude less. With one exception (trichloroethene), all groundwater concentrations were at least two orders of magnitude below available guidance or screening values. This short communication highlights information gaps in understanding the hydrologic connectivity between UIBs and receiving groundwater and potential consequent contaminant transport to the subsurface from varying climatic conditions.

**Practitioner Points:**

- Urban stormwater contains organic contaminants including pharmaceuticals, pesticides, and semi‐volatile organic compounds that may be transported to groundwater via infiltration.
- In general, fewer contaminants were detected in groundwater and at lower concentrations, compared with urban stormwater runoff.
- Trace organic contaminant concentrations in groundwater were much lower than drinking water guidance/screening values.

## Graphical abstract

Stormwater runoff is a source of trace organic contaminants, but the implications of contaminant loading to receiving groundwaters from infiltration practices are rarely studied. Results from our pilot study indicate that although relatively few contaminants were detected in groundwater near underground infiltration basins (UIBs), many questions are still unanswered, and more detailed studies would be beneficial to understand the implications of stormwater infiltration.

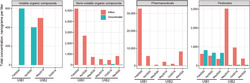

## INTRODUCTION

The management of stormwater runoff is a challenge for urban environments (NRC, 2009). Receiving waters are subject to pulses of flow and contaminants that can be harmful to biota (Howitt et al., 2014; Jefferson et al., 2017). One way to reduce direct stormwater runoff into surface waters is to implement best management practices (BMPs) that promote infiltration (Marsalek et al., 2007). Infiltration practices are used as a tool to reduce the volume of stormwater runoff originating from impervious surfaces to comply with local and regional regulations. Infiltration can be especially advantageous for communities that rely on groundwater for drinking water or other beneficial uses. However, because of human activities in urban environments, stormwater runoff is a pathway for contaminants (e.g., pesticides, pharmaceuticals, metals, and nutrients) to the environment (Fairbairn et al., 2018; Masoner et al., 2019). Despite this role as a potential conduit for contaminants, stormwater infiltration is increasingly being implemented, often with little effort to monitor receiving groundwater to characterize how it may be influenced by infiltrated stormwater.

Routine monitoring conducted by local entities in the Minneapolis‐St. Paul metropolitan area, Minnesota, USA, indicates a diversity of contaminants in urban stormwater at varying concentrations and indicates high concentrations of nutrients and metals, especially in winter and spring months (Capitol Region Watershed District, 2016; Minneapolis Park and Recreation Board, 2018a, 2018b). Recently, trace organic contaminants (TrOC), such as pesticides and pharmaceuticals (contaminants that are often overlooked in stormwater), have also been documented at concentrations that span orders of magnitude (Fairbairn et al., 2018; Masoner et al., 2019). However, these data only represent raw urban stormwater flowing into BMPs or treated (via filtration through engineered media) stormwater discharging from BMPs to surface water. The data do not address potential implications of infiltrating stormwater runoff via BMPs.

Underground infiltration basins (UIBs) represent a management option in which stormwater runoff is piped directly to an underground chamber that allows the runoff to infiltrate to the subsurface. This is different from other infiltration practices because there is no opportunity for plant uptake (e.g., rain gardens) and limited biogeochemical removal (e.g., from engineered media) prior to infiltration to groundwater. We completed a pilot project to characterize the hydrologic and contaminant influence of infiltrated stormwater on local groundwater aquifer resources near two UIBs in the Minneapolis‐St. Paul metropolitan area. The data obtained from our pilot project begin to fill some of the data gaps in knowledge regarding potential implications of stormwater infiltration.

## MATERIALS AND METHODS

### Location and site information

**FIGURE 1:**
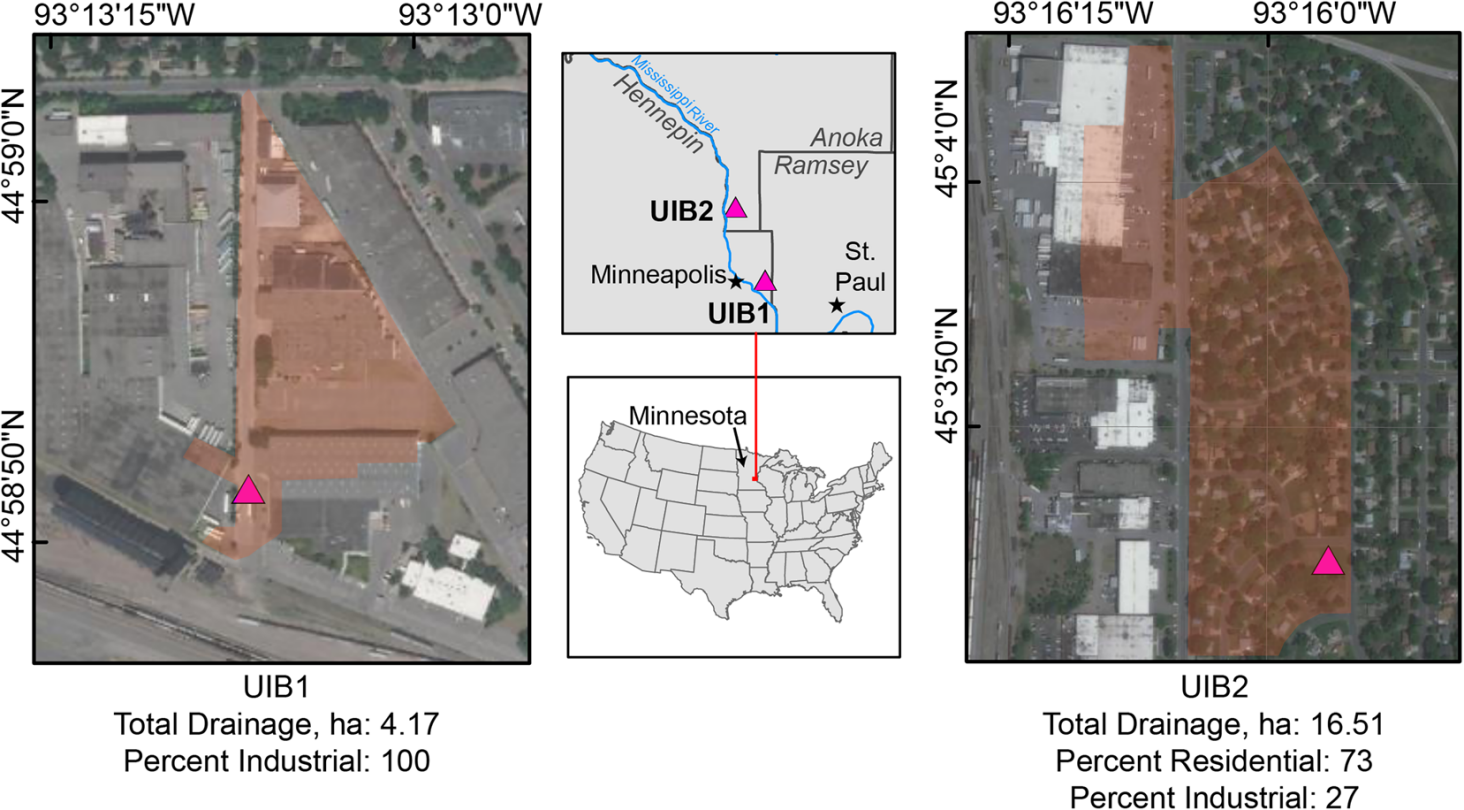
Location of underground infiltration basins (UIBs) where groundwater levels, groundwater temperature, and precipitation were monitored; and trace organic contaminants were characterized in stormwater runoff (inflow) and groundwater. Contributing area is highlighted in transparent orange. Pink diamond indicates location of UIB. Basemap credit: Esri, Maxar, GeoEye, Earthstar Geographics, CNES/Airbus DS, USDA, USGS, AeroGRID, IGN, and the GIS User Community

Two UIBs located within the Minneapolis‐St. Paul metropolitan area were selected for inclusion in the study (Figure 1). Both sites were installed as retrofits of existing infrastructure to reduce the volume of stormwater runoff that is directly discharged (via storm sewers) to the Mississippi River. Stormwater runoff is routed to a perforated culvert or gallery of pipes installed belowground at each site. Subsurface materials at UIB1 consist mostly of layers of sand/silt fill and depth to groundwater averages about 7 m below land surface. Groundwater generally flows to the southeast towards the Mississippi River. UIB1 receives stormwater runoff from a total of 5.79 ha via two inflows located on the north and south ends of the basin draining an industrial area. Subsurface materials at UIB2 consist of layers of loose fill, swamp deposits, fine alluvium, and coarse alluvium. Depth to groundwater at UIB2 averages about 4 m below land surface. Groundwater generally flows to the west towards the Mississippi River. UIB2 receives stormwater runoff from a total of 16.51 ha from a mixed residential and industrial drainage. Monitoring wells were installed on the downgradient side of each UIB, within 10 m from the edge of the underground basin. Both sites overlay the Prairie du Chien‐Jordan aquifer system. Additional details regarding site construction for both sites are provided in supplemental information.

### Groundwater and precipitation monitoring

Precipitation was measured in 15‐min intervals at the two UIB sites with a HOBO/Onset RG3 (Bourne, Massachusetts) dual tipping unheated bucket rain gage rated to 0.25 mm. Rain gages were calibrated prior to installation (Texas Electronics FC‐525 field calibration kit; Dallas, Texas) and deployed from March 17, 2020, until October 5, 2020. Submersible pressure transducers (OTT Orpheus Mini; OTT HydroMet, Loveland, Colorado) were installed in monitoring wells from November 8, 2019, until October 6, 2020. Groundwater level and groundwater temperature were measured in 15‐min intervals.

### Sample collection and analyses

Sampling was coincident with rain events that resulted in visible overland flow and increased flow (of stormwater) into the UIBs. Stormwater runoff samples were collected as grab samples at the point of entry to the UIB (inflow) and after any pre‐treatment (e.g., settling basin) by gaining access from manhole covers. Inflow was collected by pumping water to the surface using a peristaltic pump and fluorinated ethylene propylene tubing to prevent reactivity between contaminants in the sample and the sampling equipment. Because site UIB1 has two inlets, 3 L of water was collected from each inlet and composited in a polytetrafluoroethylene churn to obtain a representative sample of total water being infiltrated at the site, when possible.

Shallow groundwater samples were collected immediately after collection of inflow samples. While this method does not guarantee that the same parcel of water is being sampled, it allows for general characterization of the similarities and differences between the waters being sampled. Water was pumped to the surface using a peristaltic pump and fluorinated ethylene propylene tubing. Prior to sample collection, at least three well volumes were purged from the well and physical water‐quality properties allowed to stabilize. Additional detail about sample collection methods for environmental and quality‐assurance samples, and results for quality‐assurance samples are provided in supplemental information.

Three sets of paired (inflow and shallow groundwater) samples were collected from each site. Two sets of samples from each site were analyzed for volatile organic compounds (VOCs), semi‐volatile organic compounds (SVOCs), pharmaceuticals, and pesticides. The third set of samples was analyzed for SVOCs, pharmaceuticals, and a limited suite of pesticides. One pair of samples from each site was collected during snowmelt and the other samples were collected during precipitation events ranging from 0.25 to 4.6 cm.

All samples were analyzed at the U.S. Geological Survey (USGS) National Water Quality Laboratory (Table S2). A total of 61 VOCs were extracted from samples by active purging with helium and determined using gas chromatography/mass spectrometry methods (Connor et al., 1998). A total of 55 SVOCs were determined by extraction with methylene chloride and analysis by gas chromatography/mass spectrometry (Fishman, 1993). A total of 108 pharmaceuticals and transformation products (TPs), plus methyl‐1*H*‐benzotriazole (corrosion inhibitor included to compare performance between compounds and between methods) and atrazine, were determined using direct aqueous injection high performance liquid chromatography/tandem mass spectrometry methods (Furlong et al., 2014). Pesticides and TPs were determined using a direct aqueous‐injection liquid chromatography–tandem mass spectrometry method (Sandstrom et al., 2015).

## RESULTS AND DISCUSSION

### Environmental monitoring

**FIGURE 2:**
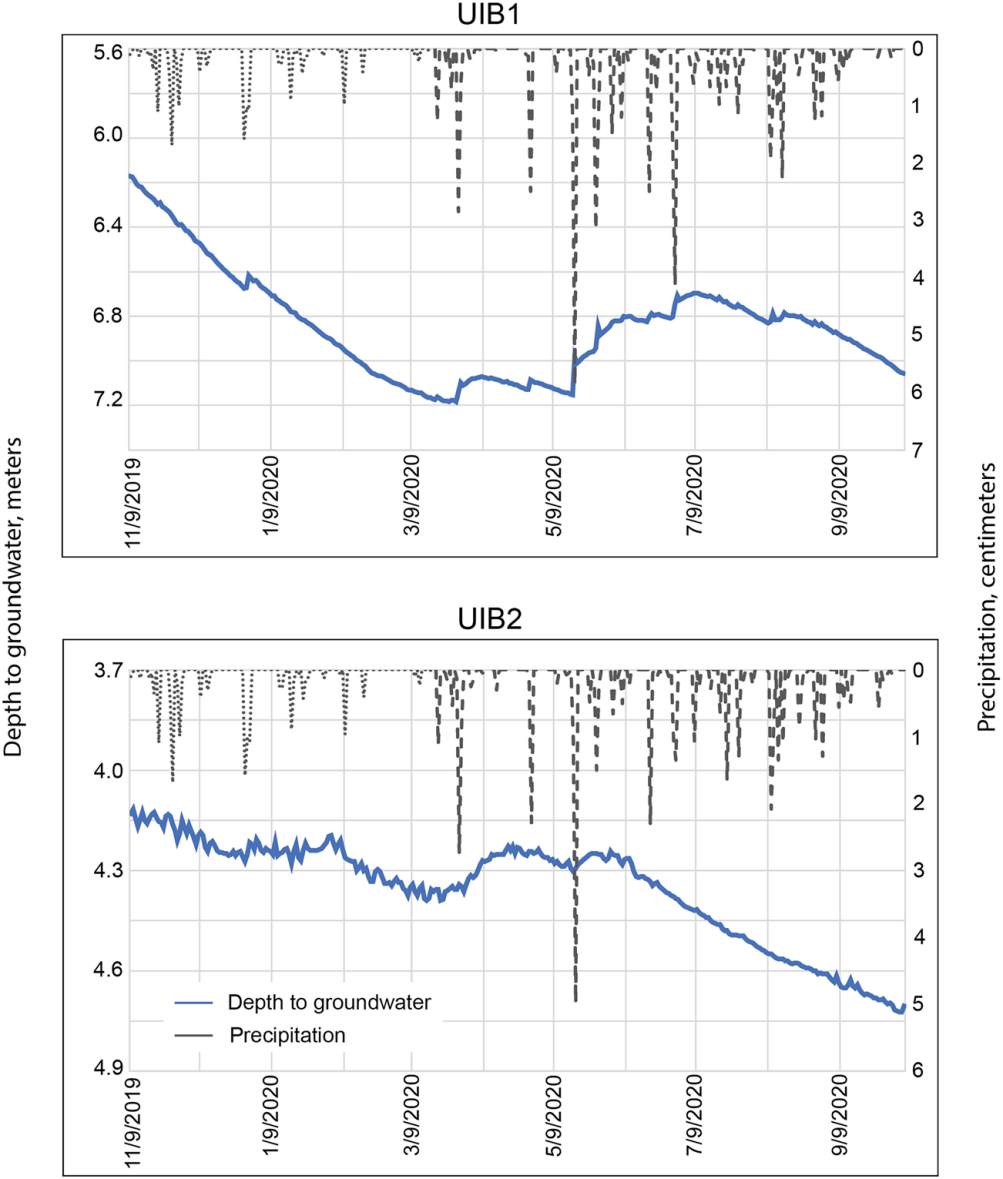
Depth to groundwater (daily mean) and precipitation (daily sum) measured near two underground infiltration basins in the Minneapolis‐St. Paul metropolitan area, Minnesota, during November 9, 2019, to October 5, 2020. *x* axis labels are given in month/day/year. Precipitation data from 11/9/2019 to 3/18/2020 (indicated by the dotted gray line) were obtained from the National Oceanic and Atmospheric Administration (2020). Precipitation measured as part of the current study (after 3/18/2020) are indicated by the dashed gray line

Groundwater level, groundwater temperature, and precipitation were monitored to gain a better understanding of how hydrologically connected the UIBs are to receiving groundwater, which can provide some insights into TrOC transport. Precipitation recorded at the two sites was very similar (Figure 2). Individual precipitation events ranged from 0.01 to 5.9 cm at both sites over the period of record. Slightly more than half of total precipitation at the sites occurred during the period of March 18 to June 18.

Depth to groundwater at UIB1 shows a system that appears to be more closely connected to precipitation (and thus infiltration), compared with UIB2 (Figure 2). Several medium to large precipitation events (e.g., March 28, April 28, May 17, May 26, and June 29; Figure 2) resulted in decreases in depth to groundwater that occurred within 24 h.

Depth to groundwater at UIB2 varied less than at UIB1, but the overall pattern was similar (Figure 2). Throughout much of the period of record, there is a slight oscillation in the depth to groundwater indicating that infiltrated water may get stored in confining layers within the subsurface and slowly released to the water table over time. This “noise” in the depth to groundwater is typical of semi‐confined aquifer systems.

### Trace organic contaminants in stormwater runoff and shallow groundwater

#### Volatile organic contaminants (VOCs)

**FIGURE 3:**
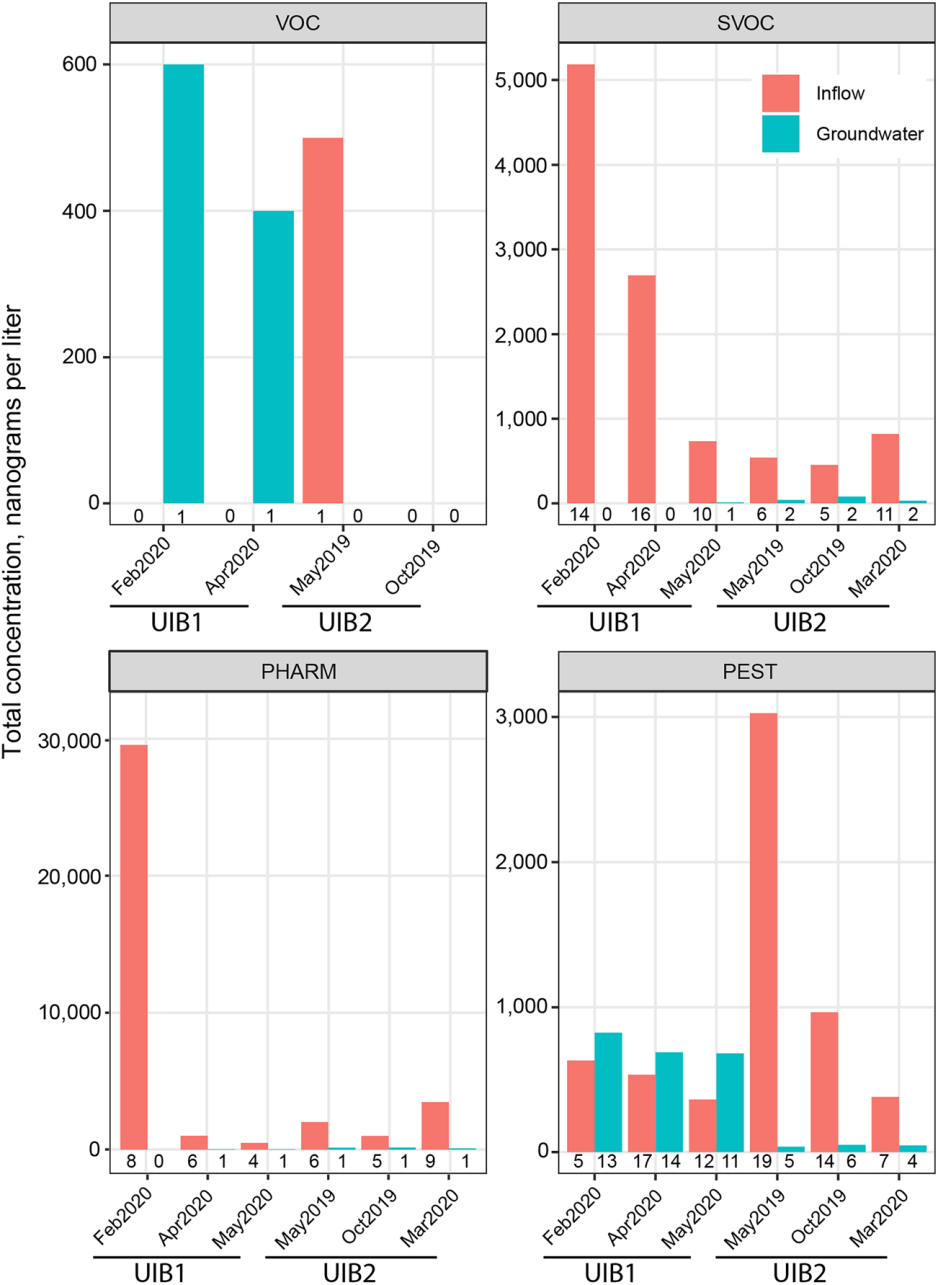
Total concentrations of trace organic contaminants in stormwater inflow and paired shallow groundwater samples collected from two underground infiltration basins (UIB) in the Minneapolis‐St. Paul metropolitan area, Minnesota, 2019–2020. Numbers below bars indicate the number of unique trace organic contaminants that were detected. Note different *y* axis scales for each class of contaminants. VOC, volatile organic compounds; SVOC, semi‐volatile organic compounds; PHARM, pharmaceuticals; PEST, pesticides

Of the 61 VOCs analyzed, two (3%) were detected in at least one environmental sample. The low detection frequency of VOCs and low detected concentrations are consistent with those reported in Minnesota groundwater (Kroening & Vaughan, 2019). Trichloroethene was detected twice in groundwater from UIB1, and toluene was detected once in inflow at UIB2 (Figure 3). Trichloroethene is used for a variety of industrial practices, is highly mobile in soils, and is relatively persistent in groundwaters (Agency for Toxic Substances and Disease Registry, 2019). Although present in surface waters, toluene can be degraded in groundwater by anaerobic microorganisms (Agency for Toxic Substances and Disease Registry, 2017), which may explain why it was not detected in any of our shallow groundwater samples (dissolved oxygen concentrations in groundwater were often <5 mg/L). A concentration of 600 ng/L for trichloroethene in one groundwater sample was slightly above the Minnesota Department of Health (MDH) drinking water guidance value of 400 ng/L (MDH, 2021; Table S4). However, trichloroethene was not detected in the associated inflow sample so more in‐depth monitoring would be insightful to fully understand the hydrologic connectivity and transport of contaminants at this site.

#### Semi‐volatile organic compounds (SVOCs)

Of the 55 SVOCs analyzed, 22 (40%) were detected in at least one environmental sample. Total sample concentrations of detected SVOCs ranged from 453 to 5183 ng/L in inflow and 10 to 80 ng/L in groundwater (Figure 3), with the highest concentrations in the snowmelt runoff sample collected at UIB1. Snowpack may act as a reservoir for SVOCs that are released back into the environment when the snow melts (Herbert et al., 2006). Concentrations of individual SVOCs ranged from 10 ng/L (isophorone) to 1280 ng/L (di‐*n*‐butyl phthalate) (Table S3). Isophorone, the most frequently detected SVOC, was detected in 83% of inflow samples and 50% of groundwater samples. Eight SVOCs were detected in at least 50% of inflow samples. However, none of the eight frequently detected SVOCs were detected in any groundwater samples. In fact, with one exception (2,4‐dichlorophenol in UIB2 groundwater on March 4, 2020), when SVOCs were detected in inflow, they were either not detected in groundwater or were detected at lower concentrations (Figure 3). Concentrations of individual SVOCs in shallow groundwater were at least a magnitude lower than available drinking water guidance values (Table S4).

#### Pharmaceuticals

**FIGURE 4:**
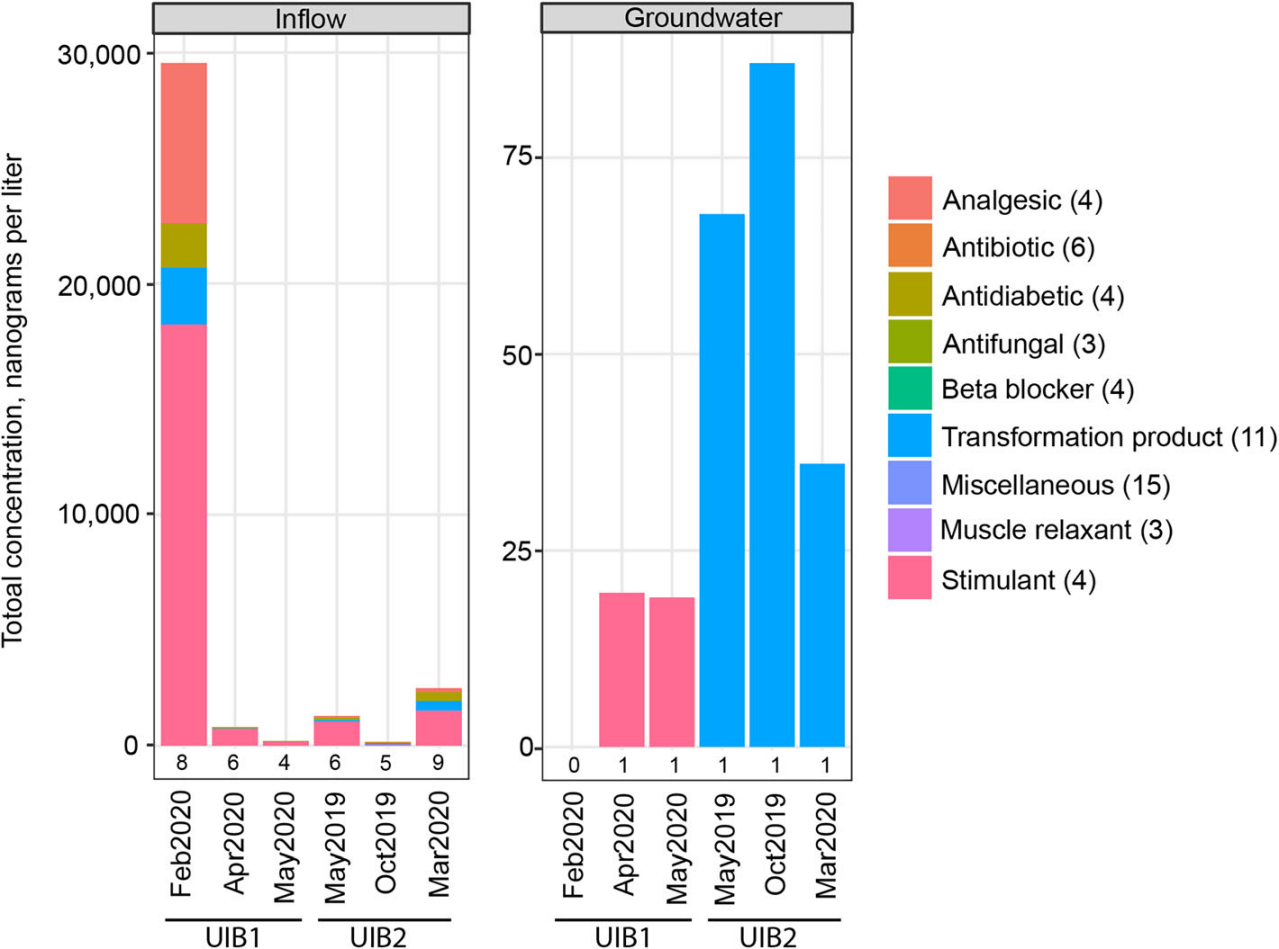
Total concentrations of pharmaceuticals, by class, in stormwater inflow and paired shallow groundwater samples collected from two underground infiltration basins (UIB) in the Minneapolis‐St. Paul metropolitan area, Minnesota, 2019–2020. Numbers below bars indicate the number of unique pharmaceuticals that were detected. Numbers in parentheses after class indicate the number of pharmaceuticals that were analyzed within that class. Note different *y* axis scales for each component figure

Of the 108 pharmaceuticals analyzed, 10 (9%) were detected in at least one environmental sample. Total sample concentrations of detected pharmaceuticals ranged from 525 to 32,055 ng/L in inflow and from 19 to 160 ng/L in groundwater (Figure 3). Concentrations of individual pharmaceuticals ranged from 0.8 ng/L (lidocaine; antiarrhythmic and anesthetic) to 13,700 (nicotine; stimulant) ng/L (Table S3). The pharmaceuticals with relatively high concentrations at UIB1 in February correspond to patterns observed in Fairbairn et al. (2018), which were attributed to antecedent conditions and little dilution. Inflow samples tended to reflect a diverse mixture of pharmaceuticals, whereas groundwater samples were dominated by nicotine and cotinine (stimulant and TP, respectively) at UIB1 and by TPs at UIB2 (Figure 4). Six pharmaceuticals were detected in at least 30% of inflow samples. However, of the frequently detected pharmaceuticals, only cotinine and nicotine were detected in groundwater samples in 50 and 17% of samples, respectively. In most instances, concentrations were lower in groundwater, compared with inflow. No guidance or screening values are available for the two pharmaceuticals (cotinine and nicotine) detected in groundwater so no comparisons could be made.

#### Pesticides

**FIGURE 5:**
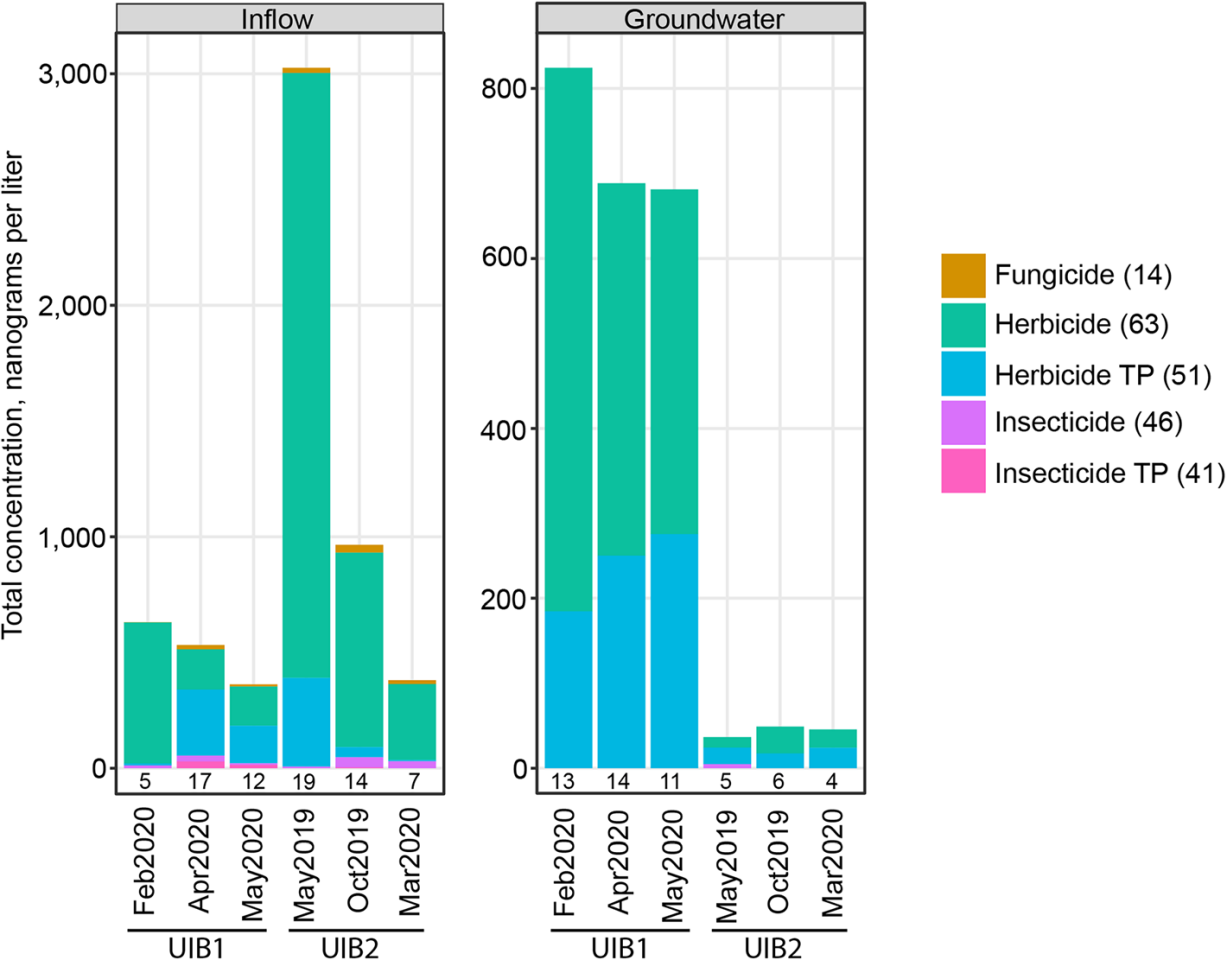
Total concentrations of pesticides, by class, in stormwater inflow and paired shallow groundwater samples collected from two underground infiltration basins (UIB) in the Minneapolis‐St. Paul metropolitan area, Minnesota, 2019–2020. Numbers below bars indicate the number of pesticides that were detected. Numbers in parentheses after class indicate the number of pesticides that were analyzed within that class. Note different *y* axis scales for each component figure. TP, transformation product

Of the 229 pesticides analyzed, 41 (18%) were detected in at least one environmental sample. Total sample concentrations of detected pesticides ranged from 363 to 3025 ng/L in inflow and 37 to 824 ng/L in groundwater (Figure 3). Concentrations of individual pesticides ranged from 0.61 (fipronil amide; insecticide TP) to 2230 (2,4‐D; herbicide) ng/L (Table S3). Twenty‐one pesticides were detected in at least 30% of inflow samples, including seven herbicides, six herbicide TPs, four fungicides, two insecticides, and two insecticide TPs. Concentrations of individual pesticides in inflow samples ranged from 0.61 (fipronil amide; insecticide TP) to 2230 (2,4‐D; herbicide) ng/L. Fourteen pesticides were detected in at least 30% of groundwater samples, including seven herbicides and seven herbicide TPs. Concentrations of pesticides in groundwater samples ranged from 0.7 (methoxyfenozide; insecticide) to 327 (bromacil; herbicide) ng/L. Our results are consistent with those of Kroening and Vaughan (2019), where herbicide TPs were more frequently detected, compared with other pesticides. Inflow samples typically consisted of a variety of pesticides and TPs, whereas groundwater samples consisted mostly of herbicides and herbicide TPs (Figure 5). Guidance or screening values are available for 16 of the pesticides that were detected in groundwater samples. All concentrations were at least one order of magnitude less than guidance or screening values (Table S4).

## CONCLUSIONS

Relatively few TrOCs were detected in groundwater during our pilot study. However, because groundwater samples were collected on the same trip as inflow samples, we did not always capture the rising limb of the groundwater hydrograph and therefore may be underestimating the presence of contaminants in receiving groundwater near these sites. Additional research would be useful to answer questions raised by this pilot study. For example, tracer tests could be used to better quantify the volume of water that monitoring wells are receiving from UIBs and determine the overall loading of infiltrated water to receiving groundwater. Monitoring TrOCs in stormwater runoff and receiving groundwater over a longer period could capture inter‐year variability which would provide an indication of how UIBs perform over varying conditions. Finally, tracking parcels of water as they flow through the UIBs could better quantify transport and degradation of TrOCs. As communities continue to implement infiltration practices as part of stormwater management plans and rely on groundwater for drinking supplies or other beneficial uses, it would be beneficial to incorporate water‐quality monitoring into management plans so that the implications of infiltrating stormwater on local groundwater resources can be more fully realized.

## CONFLICT OF INTEREST

The authors declare that they have no known competing financial interests or personal relationships that could have appeared to influence the work reported in this paper.

## AUTHOR CONTRIBUTIONS

**Sarah Elliott:** Conceptualization; data curation; formal analysis; funding acquisition; investigation; project administration; visualization. **Richard Kiesling:** Conceptualization; funding acquisition; investigation. **Andrew Berg:** Data curation; investigation. **Heiko Schoenfuss:** Conceptualization; funding acquisition.

## DISCLAIMER

Any use of trade, firm, or product names is for descriptive purposes only and does not imply endorsement by the U.S. Government.

## Supporting information


**Table S1.** Site information for underground infiltration basin inflows and monitoring wells sampled in Minneapolis‐St. Paul, Minnesota, 2019–20. All data collected for this study can be obtained by searching the U.S. Geological Survey National Water Information System (https://doi.org/10.5066/F7P55KJN) using the station numbers. Depths in meters; na, not applicable
**Table S2.** Trace organic contaminants analyzed at the U.S. Geological Survey National Water Quality Laboratory. Pesticides with an asterisk indicate those that were included in a limited pesticide analysis used for some samples. CASRN, Chemical Abstracts Serviced Registry Number; −‐, no data; na, not applicable
**Table S3.** Concentrations (nanograms per liter) of trace organic contaminants that were detected in at least one inflow or shallow groundwater sample collected near two underground infiltration basins in the Minneapolis‐St. Paul, Minnesota, 2019–2020. USGS, U.S. Geological Survey; yyymmdd, year month date; hhmm, hours minutes; °C, degrees Celsius; mg/L, milligrams per liter; μS/cm, microsiemens per centimeter; na, not applicable; <, less than; E, estimated concentration; v, sample affected by laboratory contamination; M, compound present but unquantifiable
**Table S4.** Maximum concentration of trace organic contaminants detected in groundwater and associated guidance or screening values. Concentrations and guidance/screening values are in ng/L. HRL, health risk limit; HBV, health‐based value; RAA, risk assessment advice; RAV, rapid assessment value

**Data S1.** Supporting Information

## Data Availability

Water quality, groundwater level, and precipitation data (March 17, 2020 to October 5, 2020) can be obtained by searching the U.S. Geological Survey National Water Information System (https://doi.org/10.5066/F7P55KJN) using the station numbers provided in Table S1. Precipitation data for the period of November 9, 2019, to March 18, 2020, were obtained from the National Oceanic and Atmospheric Administration NOWData database (https://w2.weather.gov/climate/xmacis.php?wfo=mpx).
