# Supplementary material for: A pilot study to assess the influence of infiltrated stormwater on groundwater: Hydrology and trace organic contaminants
Source: Water Environ Res. 2022 Feb 4;94(2):e10690. doi: 10.1002/wer.10690 (PMC9305752; doi:10.1002/wer.10690)
Supplement: Supplementary file 2 — Data S1. Supporting Information [file WER-94-0-s001.docx]

**A PILOT STUDY TO ASSESS THE INFLUENCE OF INFILTRATED STORMWATER ON GROUNDWATER: HYDROLOGY AND TRACE ORGANIC CONTAMINANTS**

Sarah M. Elliott^a^*, Richard L. Kiesling^a^, Andrew M. Berg^a^, Heiko L. Schoenfuss^b^

^a^U.S. Geological Survey, 2280 Woodale Drive, Mounds View, Minnesota, USA, 55112,

^b^St. Cloud State University, 720 Fourth Avenue South, WSB-273, St. Cloud, Minnesota, USA, 56301

*Corresponding author: [selliott@usgs.gov](mailto:selliott@usgs.gov)

**Supplementary information**

Contents

[Site information 2](#_Toc92439246)

[Sample collection 2](#_Toc92439247)

[Data preparation and analysis 3](#_Toc92439248)

[Quality-assurance results 4](#_Toc92439249)

[Volatile organic contaminants (VOCs) 4](#_Toc92439250)

[Semi-volatile organic contaminants (SVOCs) 4](#_Toc92439251)

[Pharmaceuticals 5](#_Toc92439252)

[Pesticides 5](#_Toc92439253)

[Disclaimer 5](#_Toc92439254)

[References 6](#_Toc92439255)

# Site information

Site UIB1 is an 18.9-m long, 3.05-m high, and 3.66-m wide perforated concrete box culvert that was installed beneath the existing road in 2016. A layer of aggregate was placed directly underneath the basin to facilitate infiltration to the subsurface. The depth to groundwater generally increases along a north to south gradient with water levels ranging from 4.18 to 7.41 m below land surface. A monitoring well (MW1; Table S1) was installed to a depth of 9.14 m, with a 3.05-m screened interval, on the downgradient side of and near the mid-point of the basin in October 2019.

Site UIB2 consists of an underground infiltration gallery that was installed beneath a neighborhood park turf in 2016. The infiltration gallery has a total footprint of 31.4-m length and 14.3-m width composed of a series of 2-m diameter corrugated metal pipes. Granular bedding was placed below the pipes, while crushed stone and granular material were placed around and above the pipes. A non-woven geotextile fabric encapsulates the system to prevent soil migration. A monitoring well (MW2; Table S1) was installed to a depth of 7.47 m, with a 3.05-m screened interval, on the downgradient side of and directly adjacent to the basin in January 2015. Static water level documented after well installation was 5.26 m below land surface (Minnesota Department of Health, 2020).

# Sample collection

Water temperature, specific conductivity, pH, and dissolved oxygen of stormwater inflow and groundwater were measured using a Yellow Springs Instrument (Yellow Springs, Ohio) water-quality sonde. Water quality properties were allowed to stabilize prior to sample collection. Static water levels were measured prior to removing water from the monitoring wells.

Water for volatile organic compounds (VOCs) was pumped directly into 3 or 4 (inflow and groundwater, respectively) 40-mL borosilicate vials and acidified with a 50% solution of hydrochloric acid. Water for semi-volatile organic compounds (SVOCs) was pumped directly into a 1-L glass, amber bottle. Pharmaceutical and pesticide samples were filtered through a 0.7-micron (µm) glass fiber syringe-tip filter and dispensed into 20-mL amber, borosilicate bottles. All samples were immediately chilled on ice and shipped overnight to the U.S. Geological Survey (USGS) National Water Quality Laboratory (NWQL) in Lakewood, Colorado, for analyses.

A total of three quality-assurance samples were collected: two field blanks and one replicate. Field blanks were collected by filling the appropriate sample bottles with certified organic-free blank water obtained from the NWQL. Field blanks provide information related to potential contamination associated with sample collection and processing. One replicate sample was collected sequentially after the environmental sample. Replicate samples provide information regarding variability associated with sample collection, processing, and analyses.

# Data preparation and analysis

For this report, estimated values (indicated by a ‘E’ in Table S3) were used as reported by the laboratory in data summaries. Values potentially affected by laboratory contamination (indicated by a ‘v’ in Table S3) were treated as non-detects for data summaries. Although methyl-1H-benzotriazole (corrosion inhibitor) and atrazine (pesticide) are included in the pharmaceutical analytical method, these compounds were not used in pharmaceutical summary statistics. Summary statistics such as detection frequency, minimum concentration, and maximum concentration were calculated for contaminants that were detected in at least one sample.

Environmental data were compared against field blank and replicate sample data. If a contaminant was detected in a field blank, environmental concentrations were considered affected by contamination and not used in summaries if they were less than 10 times the blank sample concentration. When a contaminant was detected in both the environmental and corresponding replicate sample, the relative percent difference (RPD) was calculated as a measure of variability between the samples. The RPD was calculated as the absolute difference between the two concentrations divided by the average concentration and multiplied by 100.

When available, environmental concentrations in groundwater were compared against the lowest chronic Minnesota Department of Health guidance value (Minnesota Department of Health, 2021a) and/or screening value (Minnesota Department of Health, 2021b; Minnesota Department of Health, 2021c) to provide context. Only groundwater concentrations were compared because these data more closely represent what could potentially be reaching groundwater and surface water resources that are used as drinking water sources (in the context of our study).

# Quality-assurance results

## Volatile organic contaminants (VOCs)

Two VOCs (dichloromethane, 200 ng/L; trichloromethane, 300 ng/L; table S2) were detected in a field blank sample collected on April 28, 2020, but those VOCs were not detected in any environmental samples. Toluene was detected in UIB2 inflow on May 27, 2019, at 500 nanograms per liter (ng/L; figure 3), in both the environmental and replicate samples (0% RPD).

## Semi-volatile organic contaminants (SVOCs)

No SVOCs were detected in either field blank. The RPD between environmental and replicate samples ranged from 0 (pyrene) to 60% (isophorone). Only two SVOCs had mismatched detections in environmental versus replicate samples: fluorene and 2,4,6-trichlorophenol. In both instances, the environmental sample concentrations were either estimated (below the reporting level) or slightly above the reporting level.

## Pharmaceuticals

No pharmaceuticals were detected in either field-blank sample. Detections of pharmaceuticals in environmental and replicate samples were consistent (i.e. there were no instances when a pharmaceutical was detected in one sample but not the other). The RPD between environmental and replicate samples ranged from 0.16% (nicotine; recreational stimulant) to 14.4% (acetaminophen; analgesic and antipyretic).

## Pesticides

No pesticides were detected in either field-blank sample. The RPD between environmental and replicate samples ranged from 0.81% (propazine; herbicide) to 23% (2-hydroxy-4-isopropylamino-6-amino-s-triazine; herbicide degradant). Propiconazole (fungicide) was the only pesticide that was not detected consistently between the environmental and replicate samples. It was detected at a concentration (7.76 ng/L) slightly above the reporting limit (6 ng/L) in the environmental sample.

# Disclaimer

Any use of trade, firm, or product names is for descriptive purposes only and does not imply endorsement by the U.S. Government.

# References

Minnesota Department of Health. (2020). Minnesota Well Index (MWI).

<https://www.health.state.mn.us/communities/environment/water/mwi/index.html>

Minnesota Department of Health. (2021a). Human health-based water guidance table. Accessed March 10, 2021.

<https://www.health.state.mn.us/communities/environment/risk/guidance/gw/table.html>.

Minnesota Department of Health. (2021b). Rapid Assessments for pesticides. Accessed April 15, 2021.

<https://www.health.state.mn.us/communities/environment/risk/guidance/dwec/rapidpest.html>.

Minnesota Department of Health. (2021c). Rapid Assessments for pharmaceuticals. Accessed April 15, 2021.

<https://www.health.state.mn.us/communities/environment/risk/guidance/dwec/pharmproj.html>
